# The dynamics of growth cone morphology

**DOI:** 10.1186/s12915-015-0115-7

**Published:** 2015-02-11

**Authors:** Geoffrey J Goodhill, Richard A Faville, Daniel J Sutherland, Brendan A Bicknell, Andrew W Thompson, Zac Pujic, Biao Sun, Elizabeth M Kita, Ethan K Scott

**Affiliations:** Queensland Brain Institute, The University of Queensland, St Lucia, Queensland, Australia; School of Mathematics and Physics, The University of Queensland, St Lucia, Queensland, Australia; School of Biomedical Sciences, The University of Queensland, St Lucia, Queensland, Australia

**Keywords:** Axon guidance, Neurite growth, Neural development, Eigenshape analysis, Shape analysis, Brain morphometry, Oscillations, Microtubules

## Abstract

**Background:**

Normal brain function depends on the development of appropriate patterns of neural connections. A critical role in guiding axons to their targets during neural development is played by neuronal growth cones. These have a complex and rapidly changing morphology; however, a quantitative understanding of this morphology, its dynamics and how these are related to growth cone movement, is lacking.

**Results:**

Here we use eigenshape analysis (principal components analysis in shape space) to uncover the set of five to six basic shape modes that capture the most variance in growth cone form. By analysing how the projections of growth cones onto these principal modes evolve in time, we found that growth cone shape oscillates with a mean period of 30 min. The variability of oscillation periods and strengths between different growth cones was correlated with their forward movement, such that growth cones with strong, fast shape oscillations tended to extend faster. A simple computational model of growth cone shape dynamics based on dynamic microtubule instability was able to reproduce quantitatively both the mean and variance of oscillation periods seen experimentally, suggesting that the principal driver of growth cone shape oscillations may be intrinsic periodicity in cytoskeletal rearrangements.

**Conclusions:**

Intrinsically driven shape oscillations are an important component of growth cone shape dynamics. More generally, eigenshape analysis has the potential to provide new quantitative information about differences in growth cone behaviour in different conditions.

## Background

Brain function depends on precisely specified patterns of wiring between neurons, and failures of wiring can compromise normal function [1-3]. This wiring develops during early life as axons grow and navigate to find their appropriate targets, often over long distances. A critical role in guiding axons to their targets during neural development is played by neuronal growth cones [4,5]. Growth cones have a remarkably complex and dynamic morphology, with their shape changing on the timescale of minutes [6-8]. *In vivo* some of these shape changes appear related to the position of the growth cone along its trajectory, with more complex morphology at choice points [9-14] suggesting that shape changes play an important role in guidance. However, previous morphological analyses of growth cones have been largely driven by human judgement regarding important shape dimensions, rather than these dimensions being determined directly from the data.

The most prominent features of growth cone structure are filopodia and lamellipodia. Filopodia can be quantified in terms of their number, positions, angles and lengths, while a simple measure of lamellipodial extent is the total area of the growth cone. One way of quantifying the shape of a growth cone at each moment is therefore to provide a list of these quantities, which for a typical growth cone with say five filopodia would consist of 21 numbers (two for the position coordinates and one each for angle and length for each filopodium, plus total area). While such a quantification can be useful, it clearly has significant limitations. First, it relies on time-lapse imaging of a resolution sufficient to resolve all individual filopodia, which can be difficult to achieve for dynamic growth cones for long periods of time, especially *in vivo*. Second, despite its length this list still ignores many obvious characteristics of growth cone shape, such as the shape of the lamellipodia. Third, the formulation of this list takes no account of the statistical structure of the actual data: it is driven by human intuition rather than an objective dissection of where the most variance of growth cone shape actually lies.

Here we therefore take a different approach to quantifying growth cone shape. The approach is based on principal component analysis (PCA), a well-known mathematical method for revealing the dimensions of a dataset which have the most variance. The application of PCA in the context of shapes is often called eigenshape analysis [15]. Each shape can be parameterised by the coordinates of a set of points, usually placed around the perimeter of the shape. This vector of coordinates can be represented as a point in a high-dimensional space, so that a set of shapes is represented by a cloud of points in that space. PCA rotates the axes of this space so that the axes are now ordered in terms of the variance in the data they explain. This reveals the directions (dimensions) through this cloud of points along which there is maximum variance, i.e., along which the cloud of points is most spread out. The first few principal components or eigenshapes then represent the most important shape dimensions, which can be seen as the fundamental building blocks of the set of shapes in question. On a cellular scale, eigenshape analysis has previously revealed important information about the shapes of keratocytes [16] and *Dictyostelium* [17]. It has also proved to be an extremely useful data analysis tool in domains as diverse as *Caenorhabditis elegans* locomotion [18], computer vision [19], palaeontology [20], botany [21] and musical instrument design [22]. Here we use eigenshape analysis to reveal the basic building blocks of growth cone morphology, previously unknown properties of how growth cone shape evolves through time, and new insights into the relationships between growth cone shape, chemotactic responses and forward movement. We then show that a simple computational model of shape changes based on dynamic microtubule instability can quantitatively reproduce the characteristic timescales present in the data.

## Results

### Growth cone eigenshapes

**Figure 1:**
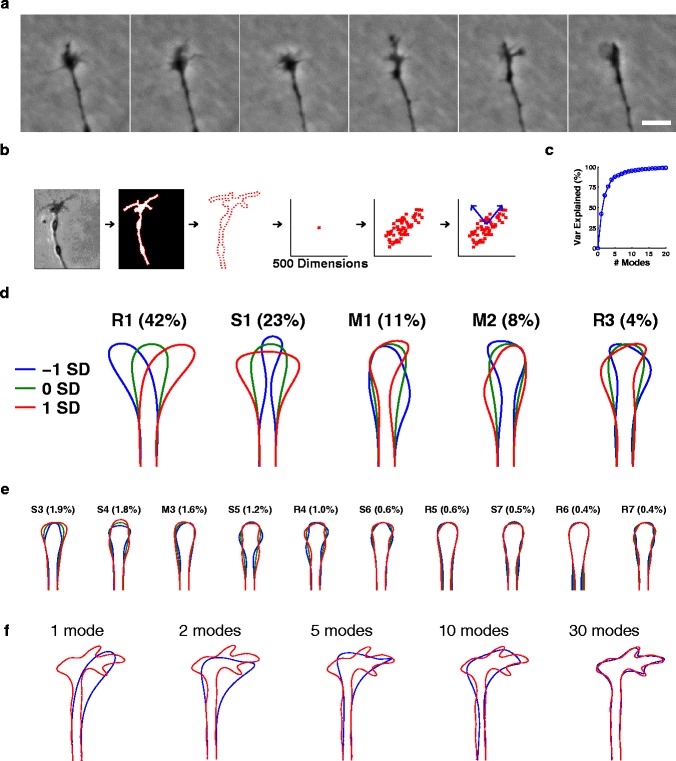
**Five significant shape modes explain 86% of the variance in growth cone shape.** **(a)** Typical sequence of frames (here 2 min apart) from a time-lapse movie of an SCG growth cone *in vitro*. Scale bar: 10 µm. **(b)** Schematic of the steps involved in the eigenshape analysis to extract the shape dimensions that capture the most variance: outline capture, parameterisation of outline by 250 evenly spaced points, principal component analysis of the resulting 500-dimensional space. Scale bar: 10 µm. **(c)** Variance explained as a function of number of mode shapes for the *in vitro* (no gradient) dataset (see Table 1). **(d)** The significant modes and their variance explained, shown as the mean shape plus the shape one standard deviation in each direction along the shape axis. Our naming convention for each mode is that the letter represents the type of symmetry, while the number is used to distinguish between different R/S/M modes. M1 and M2 approximately represent linear combinations of shapes R2 and S2 (see later). Note that all fine details (for instance, relating to filopodia) occur with a fairly random distribution around the growth cone, and are thus smoothed out once the dataset of images is appropriately large. **(e)** Higher-order shape modes and their variance explained. It is remarkable that the split between R and S symmetry persists across many higher-order modes. M3 could be arising here as an attempt to explain slight asymmetries in the underlying data. M modes in pairs, such as M1 and M2 in (c), can sometimes be understood as a linear combination of an R mode and an S mode. This occurs because when two modes have similar eigenvalues, any two orthogonal directions in that two-dimensional subspace can appear in the principal component decomposition. **(f)** Illustration of shape reconstruction using different numbers of modes. The red curve is the traced outline of a growth cone at one instant, and the blue curve is the best reconstruction of this shape given the specified number of eigenshape modes. Higher modes provide additional levels of detail for reproducing the true shape, but each individually only reproduces a tiny proportion of the variance across the full dataset. M, mixed; R, reflective; S, symmetric; SCG, superior cervical ganglion; SD, standard deviation; var, variance.

To generate a database of growth cone shapes we first made time-lapse movies of growth cones from neonatal rat superior cervical ganglion neurites (*n*=163) growing *in vitro* for 2 to 8 h (mean 2.6 h) at 15 s to 1 min intervals (see Methods, Table 1 and Figure 1a). From these we determined characteristic growth cone shapes using eigenshape analysis, i.e., PCA in the space of shapes for the dataset [15] (Figure 1b). The outline of each growth cone in each frame (*n*=25,461) was automatically extracted, and parameterised by 250 evenly spaced points. The vector of 500 numbers (250 coordinate pairs) representing each outline can be represented as a point in a 500-dimensional space (approximately 25,000 points in total for this dataset). PCA was then applied to extract the directions in the shape space that captured the largest proportion of the variance. The Bayesian information criterion (BIC) [23], which trades off variance explained versus model complexity (number of principal components retained), can be used to determine objectively the number of these dimensions that capture most of the variance of the set of shapes. According to this criterion, the optimal model retained only the top five components, which captured 86% of the variance in growth cone shape (Figure 1c). We henceforth refer to these as the significant modes.

**Table 1:** **Summary of datasets used**

	**Cell type**	**Time step**	**Duration**	**Movies**	**Frames**
*In vitro* (no gradient)	Rat SCG	15 s to 1 min	2 to 8 h	163	25,461
Pipette assay	Rat SCG	1 min	1 h	191	11,801
*In vivo*	Zebrafish RGC	1 to 10 min	1 to 24 h	27	2,249

Time step is the frequency of image capture. Each movie is for a different growth cone. RGC, retinal ganglion cell axons (approximately 2 days post fertilisation); SCG, superior cervical ganglion axons (early postnatal).

**Figure 2:**
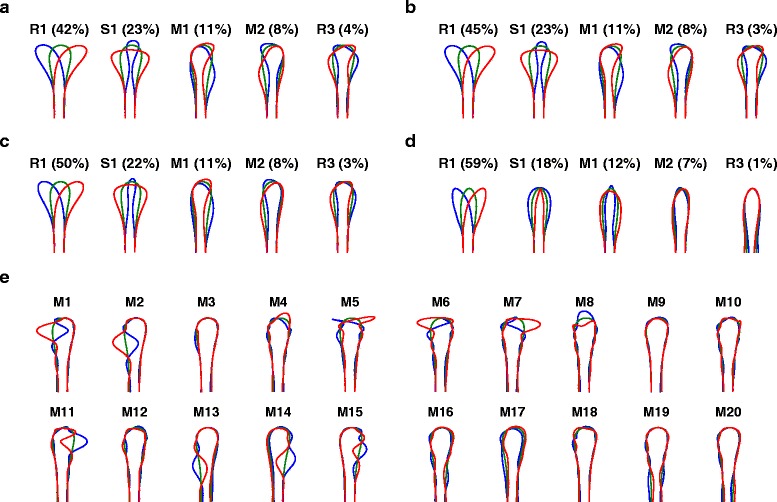
**Mode shape robustness to number of points used to parameterise the outline, and independent components analysis.** **(a)** 500 points: shapes and variance explained are the same as for Figure 1 with 250 points (five significant components). Using 1,000 points also produced identical results (data not shown). **(b)** 100 points: shapes are the same, but variance explained is slightly different (four significant components). **(c)** 50 points: both shapes and variance explained are now noticeably different (three significant components). **(d)** 25 points: shapes and variance explained are now very different (two significant components). Thus 250 points is a sufficiently detailed parameterisation such that no further changes in shape or number of significant components occur as this number is increased. **(e)** 20 independent components were found for the *in vitro* (no gradient) dataset using the FastICA algorithm [24]. Ordering is arbitrary. Some of these components (e.g., M5 to M7) appear to be trying to represent individual filopodia.

These shape modes split into three types, which we term reflective (R), symmetric (S) and mixed (M) modes (Figure 1d). For R modes, the shapes corresponding to equal positive and negative movements along the shape axis are approximately reflections of each other, with the principal R mode (R1) representing bending of the growth cone. S modes instead have approximate symmetry about their midline for all positions along the shape axis, with S1 representing spreading of the growth cone. M modes have neither of these properties, but may represent a linear combination of R and S modes (see Figure 1). The split between these different types continues for the higher-order modes (Figure 1e). The number following R, S or M refers to the logical sequence for each type of mode. This is most clear for the R modes: R1 displays one bend, R2 (see later) displays two bends, R3 displays three bends, and so on. The modes were robust to the number of points used to define the outline, provided this exceeded approximately 200 (Figure 2a–d). Thus, just five characteristic shape modes capture most of the variance in this set of growth cones, and these modes describe distinct features of growth cone morphology.

Taken individually, none of these mode shapes explicitly represent filopodia. No information has been lost overall, however: the axes of the space have simply been rotated, and adding together a sufficient number of mode shapes will perfectly reconstruct the original shape to any desired level of accuracy (Figure 1f). Rather, the analysis has determined that choosing a few axes that represent individual filopodia explicitly does not capture the largest amounts of variance in the data. It is easy to see intuitively why this would be the case. To construct a filopodium at a particular location using the PCA axes requires the addition of many higher-order modes, each of which by itself only explains a very small amount of variance across the whole dataset. Individual filopodia are sparse, in the mathematical sense that most of the time there is no filopodium at a particular position, but when it is present it is represented strongly. We therefore also performed an independent component analysis [24], which is well suited for extracting sparse structure in data. As expected, this produced mode shapes hinting at filopodia-like structures (Figure 2e). However, these independent component modes are less useful for capturing general patterns of overall shape [15], and we therefore focused on eigenshape analysis.

**Figure 3:**
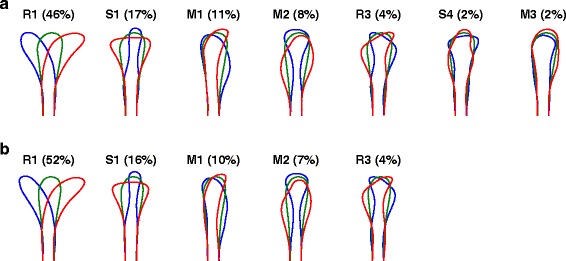
**Mode shapes are similar on a glass substrate.** All other *in vitro* data used a plastic substrate. To confirm that the higher level of image quality available using a glass substrate (e.g., greater detail regarding filopodia) did not affect our overall conclusions, we also performed time-lapse imaging of rat SCG axons growing in glass-bottomed dishes (no gradient, ten movies, 2,325 frames). **(a)** In this case there were seven significant mode shapes, but their form was similar to those observed on a plastic substrate (Figure 1). **(b)** Eigenshapes from the same dataset with artificially degraded image quality are very similar (in this case there were five significant modes). This is because all fine details (for instance relating to filopodia) occur with a fairly random distribution around the growth cone, and are thus smoothed out once the dataset of images is appropriately large. SCG, superior cervical ganglion.

To maximise throughput, we used a plastic substrate and relatively low magnification imaging for our experiments. Could the significant eigenshapes derived from these data be missing key features of growth cone shape that would become apparent from analysis of higher-quality images? To address this we also performed experiments on a glass substrate (ten movies, 2,325 frames), which allowed, for instance, clearer visualisation of filopodia. In this case there were seven significant mode shapes, but their form was similar to those observed on a plastic substrate (Figure 3a). From this dataset we also generated degraded growth cone outlines to match approximately the quality of outlines available using the plastic substrate. The resulting modes shapes were very similar (Figure 3b; in this case there were five significant modes). Thus the leading shape modes are not very sensitive to the level of detail at which the growth cone outlines are captured, making this form of quantification suitable for a much broader range of imaging regimes than quantifications relying on precise identification of individual filopodia.

### Growth cones oscillate

**Figure 4:**
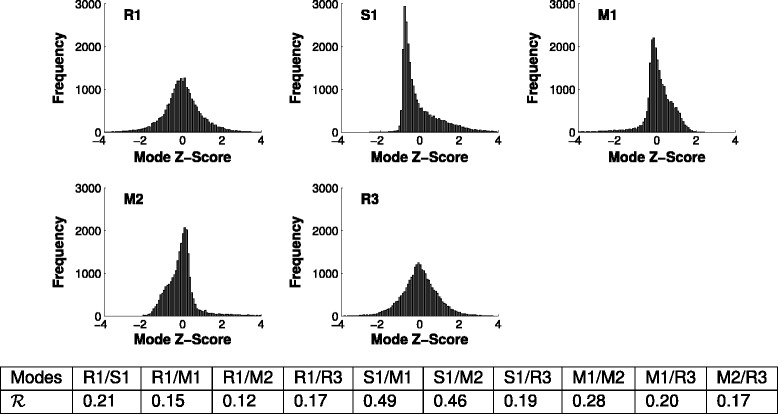
**Mode score distributions and relations across the** ***in vitro*** **(no gradient) dataset.** R (reflective) modes are symmetrically distributed around the mean shape, while S (symmetric) and M (mixed) modes have skewed distributions. This is because they contain a ‘thinness versus fatness’ component, which has a hard lower limit. We also examined the joint distributions of pairs of modes. No linear correlations were expected since the shape modes are by definition orthogonal, and no obvious structure was visually apparent. However, a statistical test for nonlinear relationships [25] showed some dependencies between modes, particularly S1/M1 and S1/M2 (values shown in table; the measure varies between 0 and 1, with 0 indicating statistical independence).

Each growth cone outline can be projected onto each shape mode to give a set of mode scores. These measure the degree to which each shape is represented by each outline, i.e., the position of the growth cone shape along that shape axis. The overall mode score frequency distributions are shown in Figure 4. As expected the R mode projections have roughly symmetric frequency distributions, while M modes have slightly asymmetric distributions, and S modes have highly asymmetric distributions. There are no linear relationships between the distributions of pairs of mode scores since, by definition, PCA dimensions are orthogonal. A statistical test for nonlinear relationships [25] showed some dependencies between modes, though plotting pairs of mode scores against each other did not reveal any obvious patterns (data not shown).

**Figure 5:**
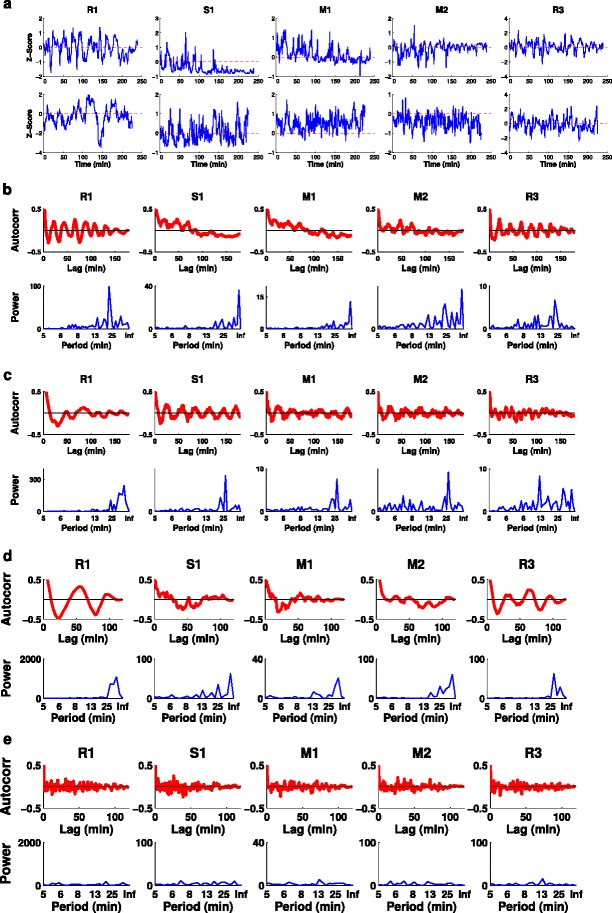
**Mode shapes oscillate over time.** **(a)** Variations in mode scores over time for the top five modes for two example growth cones. Note the clear oscillations, particularly for R1 (reflective mode 1) in the top row and S1 (symmetric mode 1) in the bottom row. **(b,c)** Autocorrelation and Fourier power spectra for all significant modes from the above two growth cones. **(d)** Analysis of oscillations for another example growth cone, this time showing much longer-period oscillations in R1. **(e)** Shuffle control for the growth cone from panel (d). Randomly shuffling the positions of all frames within the movie destroys oscillations. This is seen most clearly by the presence of very weak power, spread across many frequencies, in the Fourier power spectrum. We also shuffled all frames across the entire dataset 10,000 times, and calculated the mean total power and peak power. In all 10,000 cases these mean power values were less than for the unshuffled data. Autocorr, autocorrelation.

**Figure 6:**
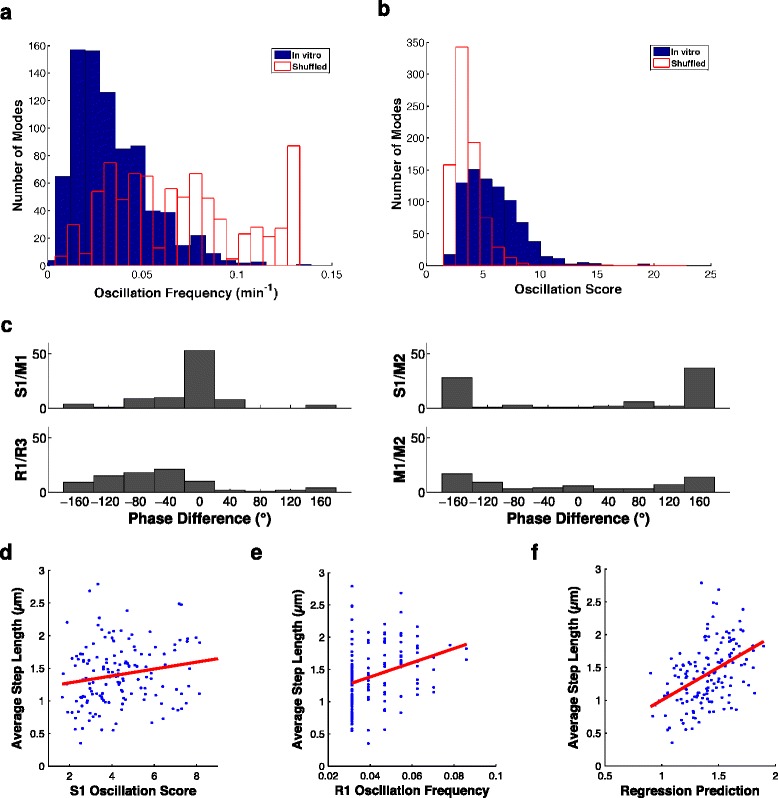
**Properties of oscillations.** Oscillations are predictive of movement. **(a,b)** Distribution of strongest oscillation frequencies (a) and scores (b) across the *in vitro* dataset (blue). Also shown are the corresponding distributions for the shuffled controls (red). The mean of the oscillation scores is 6.0, while for the shuffled controls it is 3.5 (*P*<0.001). **(c)** Phase relationships between shape mode oscillations. Frequency distributions of phase relationships are shown for pairs that showed consistent relationships for the five significant modes in the *in vitro* (no gradient) dataset. The lack of consistent phase between R (reflective) and S (symmetric) modes is not surprising, due to their fundamentally different symmetry properties. If, for instance, R1 and S1 were consistently in phase, it would mean that growth cones bending left were consistently fatter (or thinner) than growth cones bending right. **(d,e)** Average step length is correlated with mode S1 oscillation strength (see Methods) (d) and mode R1 oscillation frequency (e). **(f)** Strengths and frequencies of all significant modes together are predictive of average step length. See Tables 2, 3 and 4.

We then examined how mode scores varied through time for each individual growth cone (Figure 5a). These revealed strong hints of periodic behaviour. For instance in the top left panel of Figure 5a, the R1 mode score varies regularly between positive and negative values, indicating a periodic alternation in the growth cone shape between bending left and bending right. To detect periodic patterns quantitatively, we used the autocorrelation and Fourier power spectrum of mode scores. Oscillations in mode scores were common in all significant modes (examples shown in Figure 5b,c,d). Similar oscillations were seen on a glass substrate (data not shown). Oscillation strength and frequency for each mode were quantified using a modified version of the method of [26] (see Methods). The distributions of oscillation strengths (hereafter oscillation scores) and frequencies for all significant modes (*n*=5×163=815) are shown in Figure 6a,b. The mean oscillation frequency was 0.0338 ± 0.0197 min ^−1^ (mean ± standard deviation), corresponding to a mean period of 30 min. The mean oscillation score was 6.0 ± 2.6. R1 oscillations were on average significantly stronger than oscillations in other modes (*P*<0.03, Wilcoxon rank-sum tests). We used shuffled controls to demonstrate that these oscillations were not simply an artefact of our analysis methods (example in Figure 5e, histograms in Figure 6a,b). The mean shuffled frequency was 0.0754 min ^−1^, and the mean shuffled score was 3.5. *t*-tests comparing the real and shuffled distributions gave *P* values of 10^−76^ for frequencies and 10^−107^ for scores. Different modes for the same growth cone sometimes oscillated at the same frequency, and sometimes at different frequencies (see examples in Figure 5), with the former case revealing a range of phase relationships (Figure 6c). Overall, we conclude that growth cone shape oscillates on an average timescale of 30 min.

**Table 2:** **Correlations between mode scores**

**Mode score**							**Unsigned mode score**				
		**R1**	**S1**	**M1**	**M2**	**R3**	**R1**	**S1**	**M1**	**M2**	**R3**
Step length	*r*	0.01	0.11	−0.01	0.03	−0.01	0.08	0.01	0.05	0.09	0.07
	*P*	0.13	10^−47^	0.06	0.001	0.15	10^−25^	0.28	10^−13^	10^−34^	10^−18^
Absolute bearing	*r*	−0.00	0.06	0.02	0.00	0.02	0.03	0.06	0.06	0.06	0.02
change	*P*	0.92	10^−16^	10^−4^	0.61	0.03	10^−4^	10^−13^	10^−16^	10^−17^	0.02
Bearing change	*r*	0.03	−0.00	0.00	0.00	−0.01	−0.00	−0.01	−0.01	−0.01	−0.00
	*P*	0.0002	0.80	0.95	0.72	0.24	0.54	0.14	0.07	0.37	0.84

The data are for each moment and growth cone movement over the following minute for the *in vitro* (no gradient) dataset. *r* is the Spearman correlation. Correlations for both signed and unsigned (absolute value) mode scores are shown.

**Table 3:** **Correlations between oscillation strength and period for the average step length**

**Oscillation strength**							**Oscillation period**				
		**R1**	**S1**	**M1**	**M2**	**R3**	**R1**	**S1**	**M1**	**M2**	**R3**
Average step length	*r*	0.08	0.20	0.05	0.12	0.03	−0.37	−0.15	−0.18	−0.06	−0.11
(growth rate)	*P*	0.34	0.016	0.56	0.16	0.75	10^−5^	0.073	0.024	0.45	0.19

This is for the *in vitro* (no gradient) dataset. Average growth rate (step length averaged over each movie) is most correlated with S1 oscillation strength and R1 oscillation frequency. No significant results were found for signed or unsigned bearing change (data not shown). The oscillation strength measure was designed to be accurate on narrow bands of frequency, and is known to be affected by large variations in frequency as a result of non-linear scalings in the computed power spectrum [26]. We therefore only considered oscillations with a period under 32 min (frequency over 0.031 min ^−1^).

**Table 4:** **Regression of oscillation strengths and periods for all significant modes and average step length**

		**Oscillation**	**Oscillation**	**Oscillation**
		**strength**	**period**	**strength**
				**and period**
Average step length	*R*	0.26	0.34	0.46
	*P*	0.075	0.002	10^−4^

This is for the *in vitro* (no gradient) dataset. *R* is the coefficient of multiple correlation, the multidimensional generalisation of the correlation coefficient.

### Relationship between oscillations and movement

Given the variability of oscillation strengths and frequencies about their means, we were interested in whether there were any predictive relationships between mode scores, or oscillation strengths and frequencies, and growth cone movement between frames, characterised by step lengths (distance moved) and bearing changes (angular difference in direction) between consecutive frames. Step length from time *t* to *t*+1 was weakly positively correlated with S1 mode score at time *t* (*r*=0.11, *P*=10^−47^, Spearman correlation), meaning that wider growth cones had a small tendency to take larger steps. Other mode scores were almost uncorrelated with the movement of the growth cone (Table 2), and in particular the correlation of the R1 mode score with subsequent bearing change, although significant, was very small. Thus the periodic alternation in shape of bending left versus bending right did not translate into zigzags in overall axon trajectory. Rather, a periodic sweeping left and right of the growth cone was superimposed on a steady forward motion. R1 oscillations could be seen as a way by which growth cones might systematically probe or ‘sniff’ the environment for molecular or topographical cues, sweeping out more area than they would without these oscillations.

However, oscillation strength and frequency for each movie were correlated with the movement of the growth cone averaged over all frames (Table 3). Average growth rate (step length averaged over each movie) was correlated with S1 oscillation strength (see Methods, *r*=0.20, *P*=0.016, *n*=150, Figure 6d) and R1 oscillation frequency (*r*=0.37, *P*=10^−6^, Figure 6e). Performing a linear regression of average step length against the ten variables of oscillation score and frequency for the five significant modes produced a good prediction of average growth rates (*R*=0.46, *P*=0.0002, Figure 6f, Table 4). (Note that oscillation strength and frequency are properties of an entire movie, and thus cannot be correlated with growth cone behaviour at one moment in time, such as instantaneous growth rate.) Thus, growth cones that are oscillating strongly and rapidly tend to make forward progress faster.

### Oscillations during chemotaxis

**Figure 7:**
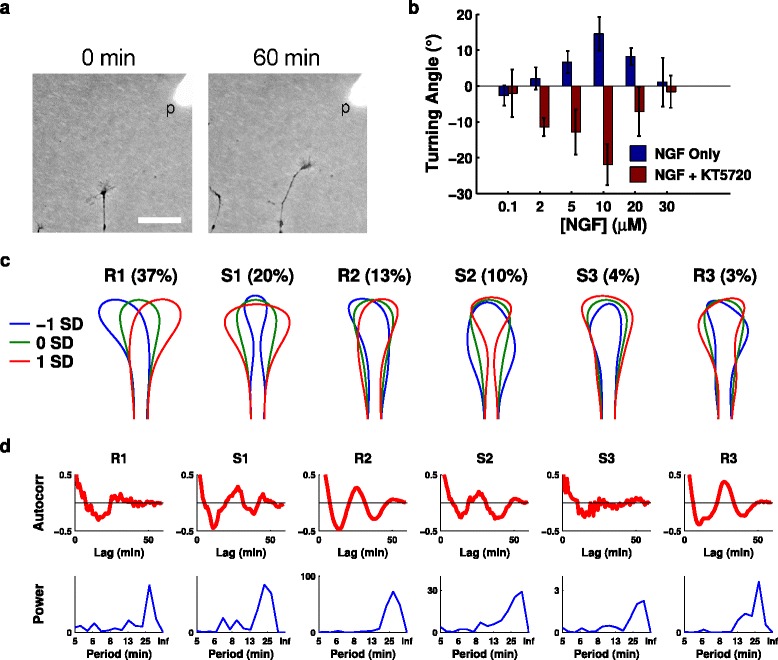
**Similar modes and oscillations are seen during chemotaxis.** **(a)** Example of growth cone moving in response to gradient produced from a pipette (p). Scale bar: 40 µm. **(b)** Chemotactic sensitivity curves (final turning angle after 60 min of growth) for both attractive and repulsive conditions. **(c)** Significant modes for pipette assay movies. The two M (mixed) modes from Figure 1c have now separated more clearly into R (reflective) and S (symmetric) modes, but otherwise the mode shapes, and their variance explained, are very similar to the *in vitro* (no gradient) dataset. **(d)** Oscillations in a representative pipette movie. NGF, nerve growth factor; SD, standard deviation.

An important mechanism by which axons are guided *in vivo* is chemotaxis. We therefore applied eigenshape analysis to determine the characteristic behaviour of a new set of growth cones as they underwent chemotactic movement in the growth cone turning (or pipette) assay for 1 h [27] (Figure 7a, Table 5). This assay produces a gradient steepness of approximately 10% across the growth cone [28]. Previously it was found that axons growing in gradients of steepness less than 1% show a bell-shaped chemotactic sensitivity curve predicted well by a Bayesian model of the chemotactic response [29]. We confirmed that a similar curve holds for steeper gradients (Figure 7b), as theoretically predicted [30]. By reducing PKA activity in the growth cone via addition of KT5720 [31-33], we also found that the chemotactic response for repulsion was approximately the mirror image of that for attraction (Figure 7b).

**Table 5:** **Summary of the pipette assay experiments**

	**NGF Only**		**NGF + KT5720**	
**[NGF]** **(µM)**	**Movies**	**Frames**	**Movies**	**Frames**
0.1	15	878	13	814
2	17	1,033	9	593
5	18	1,099	18	1,167
10	9	566	10	595
20	17	1,064	12	764
30	10	622	17	1,059
Total	88	5,376	79	4,992

[NGF] refers to NGF concentration in the pipette. There were also 26 control movies, totalling 1,547 frames. NGF, nerve growth factor.

**Figure 8:**
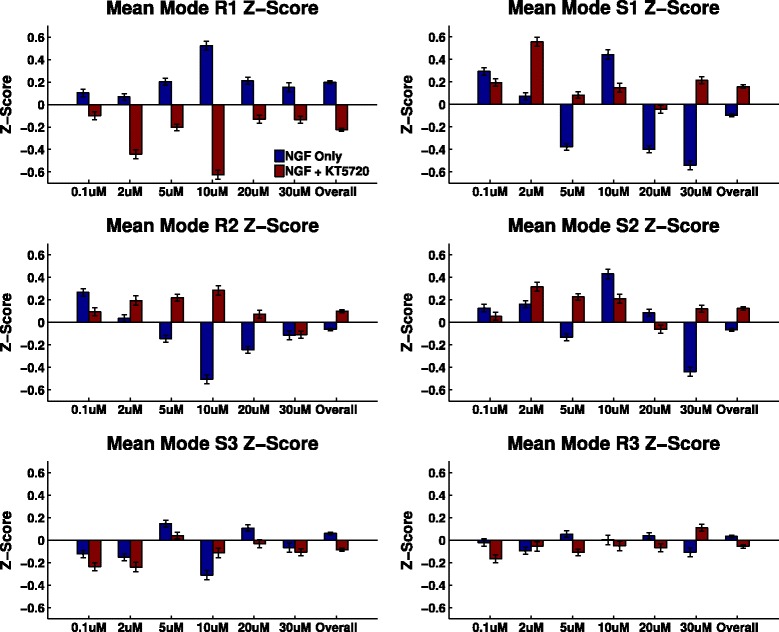
**Average mode scores as a function of NGF concentration for the pipette assay data.** These show how average growth cone shape is affected by concentration and whether the gradient is attractive or repulsive. R1 (reflective mode 1) roughly follows the chemotactic sensitivity curve (Figure 7b), while the other modes show more complex relationships. NGF, nerve growth factor.

In this dataset there were six significant eigenshape components, explaining 87% of the variance (Figure 7c). These were very similar to those we observed for the original *in vitro* (no gradient) dataset, but more clearly illustrated the split into R and S modes. These modes oscillated similarly (Figure 7d), though the short assay duration prevented observation of longer oscillations. There were no significant differences between oscillation strength and period between different gradient conditions, and no relationship was observed between final turning angle and oscillations. However, mean mode projection scores (rather than oscillation scores) varied systematically across gradient conditions, demonstrating a direct effect of chemotactic cues on growth cone shape (Figure 8, Table 6). For instance, the mean R1 mode scores roughly followed the same shape as the chemotactic sensitivity curves.

**Table 6:** **Significance values for shape differences between attraction and repulsion**

**NGF concentration (µm)**						
**Mode**	**0.1**	**2**	**5**	**10**	**20**	**30**
R1	0.00093	1.6×10^−16^	5.4×10^−20^	6.9×10^−71^	3.9×10^−11^	2.1×10^−7^
S1	0.81	1.8×10^−12^	3.4×10^−28^	0.00045	1.7×10^−12^	1.6×10^−54^
R2	0.068	0.29	5.6×10^−15^	1.5×10^−34^	8.1×10^−10^	0.96
S2	0.81	1	3.4×10^−14^	0.041	0.035	3.1×10^−38^
S3	0.00087	1	0.053	0.066	0.066	1
R3	0.053	1	0.0041	1	0.32	2.1×10^−7^

*P* values with Holm–Bonferroni correction for mean shape mode projections for NGF versus NGF plus KT5720 pipette assays (Figure 8). NGF, nerve growth factor.

In a further set of turning assay experiments (*n*=12, data not shown), we imaged growth cones for 1 h without a gradient, then applied a gradient for 1 h, to determine whether the presence of the gradient would change the properties of the oscillations for each growth cone. However, we found no significant differences.

### Eigenshapes and oscillations *in vivo*

**Figure 9:**
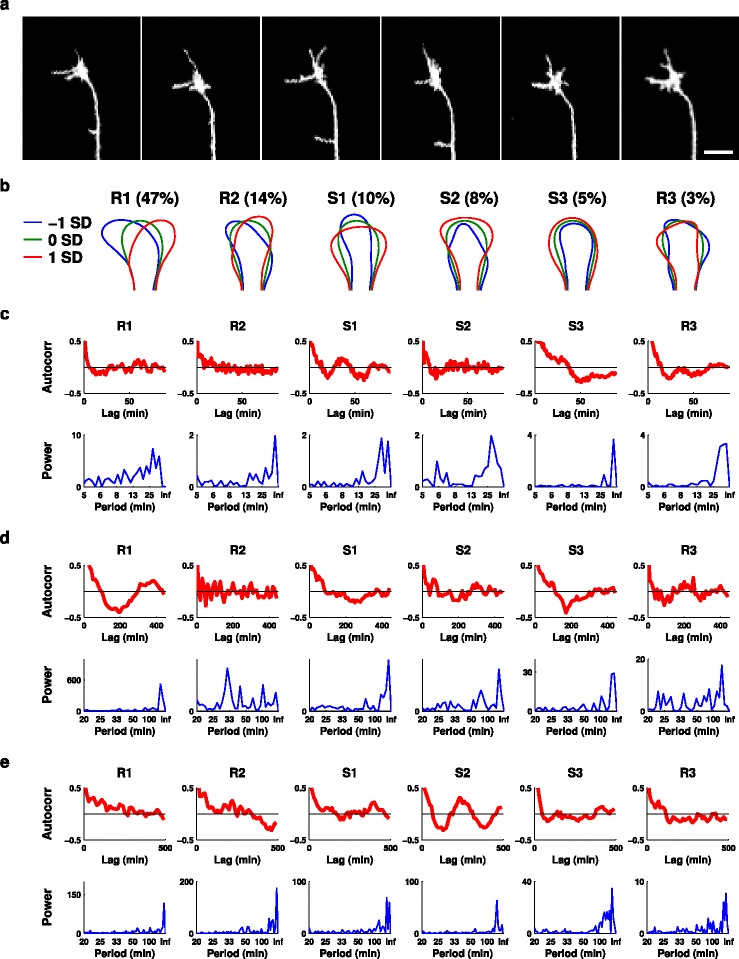
**Similar mode shapes and oscillations are also present** ***in vivo*** **.** **(a)** Typical sequence of frames 10 min apart from a time-lapse movie of a zebrafish retinal ganglion cell growth cone navigating across the tectum *in vivo*. Scale bar: 10 µm. **(b)** The significant shape modes derived from this dataset. **(c,d,e)** Mode oscillations for a 2 h movie with frames every 1 min (c), and 20 h movies with frames every 10 min (d,e). In the former case oscillations of similar period to those observed *in vitro* are seen, while in the latter cases oscillations with longer periods become apparent. Autocorr, autocorrelation; SD, standard deviation.

To investigate whether similar behaviour is observed *in vivo*, we performed time-lapse imaging, image segmentation and eigenshape analysis as before on mGFP-labelled growth cones (*n*=27) of zebrafish retinal ganglion cell axons as they navigated across the optic tectum for periods of 1 to 24 h (mean 14.5 h) (Figure 9a) [34]. This revealed six significant shape modes that were very similar to those seen for the *in vitro* no gradient and pipette datasets (Figure 9b). Oscillations in mode shapes were also qualitatively similar (Figure 9c,d,e). The mean oscillation frequency was 0.0148 ± 0.0134 min ^−1^. The difference with the *in vitro* frequency is likely at least partly due to the longer duration of the movies, which allows lower frequency oscillations to be included in the mean (see the simulations in the following section). The mean oscillation score was 6.6 ± 3.2, similar to the *in vitro* data. Correlations between oscillations and average step lengths were small (data not shown), but this dataset is much smaller than the *in vitro* dataset. Thus, the eigenshapes and oscillations displayed by growth cones as they navigate in a molecularly and structurally complex *in vivo* environment are similar to those displayed in a much simpler *in vitro* environment, arguing that these behaviours represent fundamental characteristics of growth cones rather than an *in vitro* artefact or the response to a specific environment.

### A computational model of oscillations

**Figure 10:**
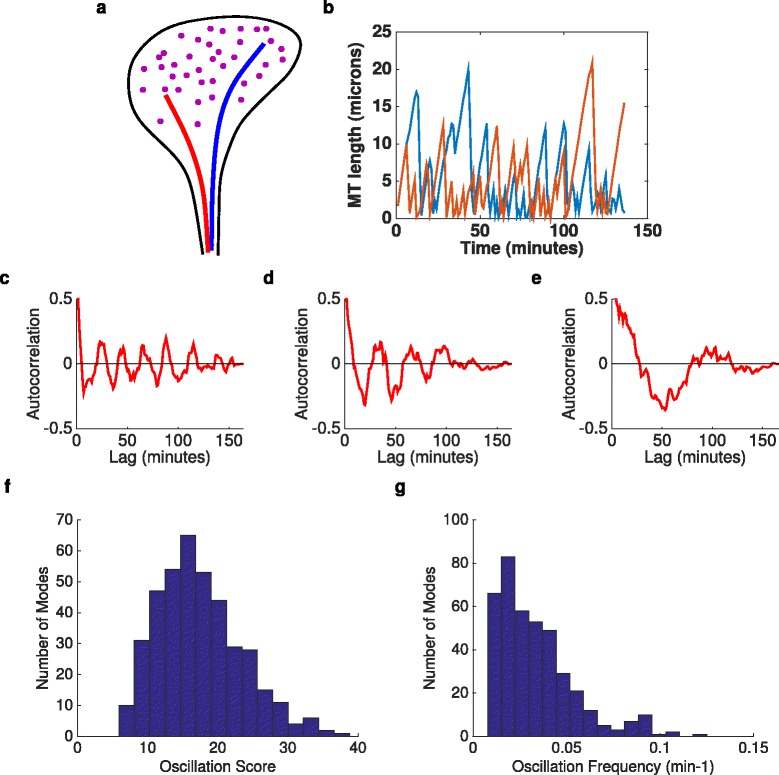
**Computational model of dynamic microtubule instability reproduces the periodicities seen experimentally.** **(a)** The model consists of two microtubules (red and blue lines) competing for a limited supply of tubulin monomers (purple dots) within the growth cone. **(b)** Lengths of both microtubules as a function of time for a typical simulation. **(c,d,e)** Autocorrelation functions for three different simulations with identical parameters but different random seeds, illustrating strong periodicity but also different frequencies at which these can occur in the model. **(f,g)** Distribution of oscillation scores (f) and frequencies (g) across 400 simulated growth cones. MT, microtubule.

What events inside the growth cone could be driving the strong periodicity in shape dynamics we have observed? Can we explain both the mean period of 30 min, and the large variability about this average? A critical component of the growth cone cytoskeleton is microtubules [35,36]. These extend from the axon shaft into the body of the growth cone, and sometimes into individual filopodia [37]. Microtubule growth is characterised by dynamic instability, whereby phases of growth are followed by catastrophic collapse, and then a return to the growth phase [38]. Walker et al. [39] constructed a computational model of this phenomenon, with parameter values constrained directly from experimental measurements. Janulevecius et al. [40] then adapted this model to show that the small volumes of cells, and thus the limited supply of free tubulin, could significantly impact on microtubule dynamics. As a minimal model of growth cone shape changes, here we consider two microtubules within a growth cone competing for the same limited supply of tubulin monomers (Figure 10a).

We assume that the extension of one of these microtubules corresponds to a shape deviation in one direction (e.g., bending of the growth cone to the right), while the extension of the other microtubule corresponds to a shape deviation in the opposite direction (e.g., bending to the left). Referring to the lengths of the two microtubules at time *t* as *l*_1_(*t*) and *l*_2_(*t*), we take the normalised difference in lengths *L*(*t*)=(*l*_1_(*t*)−*l*_2_(*t*))/(*l*_1_(*t*)+*l*_2_(*t*)) as an analogue for the projection onto an eigenshape mode. The model and parameters for each microtubule were taken directly from [40] (see Methods), with the growth cone volume assumed to be 30 µm ^3^ (i.e., roughly 6 µm in diameter and 1 µm high). The only interaction between the microtubules was via their competition for the same pool of tubulin monomers.

Growth cones (*n*=400) were simulated for the equivalent of approximately 2.6 h (the mean length of movies in the *in vitro* dataset, corresponding to 3×10^6^ events in the Monte Carlo simulation). *L*(*t*) was then subsampled to one value every minute of simulated time, the time resolution of most of our *in vitro* experimental data. As expected, *l*_1_(*t*) and *l*_2_(*t*) tended to compete with each other (Figure 10b). We found that *L*(*t*) generally showed periodic oscillations over time, qualitatively resembling the experimental data (Figure 10c,d,e). To quantify this behaviour, oscillation scores and frequencies were calculated for *L*(*t*) exactly as for the experimental data, producing distributions over the set of simulated growth cones. Oscillation scores were larger than for the experimental data, with a mean and standard deviation 17.4 ± 6.0, compared to 6.0 ± 2.6 for the experimental data (Figure 10f). This is perhaps not surprising, since there are many additional sources of noise in the data, which are not present in the model. However, surprisingly given the simplicity of the model, there was an extremely close match between the oscillation frequencies of the model and the *in vitro* data: 0.0333 ± 0.0210 min ^−1^ for the model, compared to 0.0338 ± 0.0197 for the data (*P*=0.9, *t*-test, Figure 10g). Thus, the model implicates dynamic microtubule instability as the driving force behind oscillations in growth cone shape.

The model also allows an analysis of how the duration of simulated time affects mean frequencies. Intuitively, longer durations would lead to lower average frequencies, since more distant parts of the underlying frequency distribution are now included in the average. Simulating 100 growth cones for 1.7×10^7^ events (approximately 15 h of real time, similar to the mean for the *in vivo* dataset) gave a mean frequency of 0.0176 ± 0.0124 min ^−1^, confirming this intuition. This was not significantly different to the mean oscillation frequency observed in the *in vivo* data (*P*=0.12), suggesting that the lower mean frequency observed in our *in vivo* compared to *in vitro* data is mainly due to the longer average length of these movies, rather than any fundamental difference in oscillations between *in vitro* and *in vivo* settings. This is consistent with the idea that oscillations are intrinsically driven.

## Discussion

Neuronal growth cones have one of the most dynamic morphologies of any (sub)cellular system, and this is challenging to quantify. Here we have applied for the first time to growth cones a shape analysis technique that has proved useful in many other contexts, and shown that just a few basic shape primitives capture the vast majority of the variance in growth cone shape. The form of the leading modes themselves is quite intuitive: R1 represents bending while S1 represents thinness versus fatness, whether these are a result of lamellipodial or filopodial outgrowth. By reducing shape to just the list of numbers representing the projections onto the significant modes, it becomes possible to identify patterns in the data that are not otherwise apparent. In particular, by examining how these projections evolve over time, we found that shape oscillations are a key organising principle of growth cone shape dynamics. The forward movement of growth cones has previously been reported to follow cycles of pausing and growth [41,42], and zigzag behaviour in gradients [43]. However, our morphometric [44] analysis reveals for the first time the shapes representing the most important degrees of variance in growth cone morphology (i.e., eigenshapes), how the projections of these shapes vary through time (i.e., oscillations), and how they are correlated with growth cone movement (i.e., growth cones with strong fast shape oscillations tended to extend faster). We observed the same general patterns of eigenshapes and oscillations *in vitro* and *in vivo*, suggesting that oscillations are a surprisingly robust and fundamental aspect of growth cone behaviour.

We also presented a computational model of growth cone shape changes based on dynamic microtubule instability. This model is clearly a highly abstracted version of reality, and is intended as simply a minimal model of variation along a shape axis (e.g., bending left versus bending right). It is therefore remarkable that such a simple mechanism produces an excellent match to not only the mean frequency of oscillations in the data, but also the variance in this distribution. This provides strong support for the hypothesis that the basic driver of the growth cone shape oscillations we have observed is dynamic microtubule instability in the context of competition for a limited supply of tubulin monomers. Clearly in reality many other components will be involved, most notably the actin cytoskeleton, and how these work together to determine oscillations remains to be determined.

We have examined growth cones in a relatively featureless *in vitro* environment, a chemotactic gradient, and traversing the optic tectum *in vivo*. Eigenshapes and oscillations are remarkably consistent between these different cases. This argues that these properties are intrinsic and do not represent responses to the local environment, such as a way of navigating around local obstacles, consistent with an explanation in terms of intrinsic periodicity of microtubule growth. The complete trajectories of growth cones *in vivo* often involve navigating through many stages and choice points, for instance retinal axons growing from the eye to the brain [45,46] or callosal axons finding their targets in the opposite hemisphere [47]. Eigenshape analysis provides a method for re-examining more quantitatively exactly how shape changes over such trajectories, and how it is correlated with navigational function. Whether shape oscillations change their properties over complex trajectories and how such changes might be related to the environmental cues present at each moment remain to be determined.

Our tracing of growth cone outlines was performed on relatively low-magnification phase contrast (*in vitro*) or fluorescent (*in vivo*) images, and thus fine details of some filopodia were inevitably lost. However, we have demonstrated directly that the eigenshapes we found are quite robust to image quality (Figure 3). Filopodia are not ignored in our analysis: for instance the presence of a filopodium in the right-hand side of the growth cone will show its effect by increasing the score for mode R1 (cf Figure 1f). Eigenshape analysis complements rather than replaces fine-scale analysis of filopodial dynamics, since it combines filopodial and lamellipodial structure to emphasise overall shape trends rather than fine-scale structure. Despite recent advances for fluorescently labelled growth cones [48], fine analysis of filopodia is still limited by the difficulty in obtaining large datasets: fully automated image analysis techniques have difficulty with phase-contrast imaging at this level of detail, and hand-tracing is prohibitively time-intensive. Unfortunately manual observations are not scalable [49], meaning that there is currently a growing mismatch between our ability to manipulate growth cones and to assess the effect of these manipulations on growth cone behaviour using manual techniques.

## Conclusions

Eigenshape analysis of growth cones has the potential to provide a novel quantitative understanding of the differences between growth cones in different conditions. These include the effects of the environment as axons navigate towards their targets during development, differences between initial development and regeneration, and differences between mutants and wild type. Overall this work reveals a new dimension to the understanding of the dynamic morphology of growth cones, and potentially opens up novel directions for research into understanding the biological basis of developmental brain disorders.

## Methods

All experiments were approved by The University of Queensland Animal Ethics Committee and were performed according to the National Health and Medical Research Council’s animal code of practice.

### *In vitro* imaging

Neurons from superior cervical ganglia (SCG) from P0-P4 Wistar rat pups were prepared as previously described [50]. SCGs were incubated in 0.25% trypsin (Gibco) at 37°C for 20 min and then triturated for 10 min. Cells were plated in Opti-MEM solution containing 10 µg/mL mouse laminin (Invitrogen) and 0.3 nM nerve growth factor (2.5S mouse NGF, Biosensis) and incubated overnight at 37°C in 35 mm plastic or glass-bottomed Petri dishes. Phase contrast images of growth cones were acquired at 20 × for 2 to 8 h at 1 min intervals with a Zeiss Axio Observer.

### Imaging in steep gradients of nerve growth factor

SCG neurons were prepared as above. After overnight growth, steep gradients of NGF were produced as reported previously [27,50]. Briefly, axons were positioned with their growth cones 100 µm away from a glass micropipette containing NGF, and with their direction of growth at 45° to the pipette tip. NGF was expelled at 2 Hz using 3 psi to create gradients with a 10% to 15% change in concentration across 10 µm. Phase contrast images were acquired at 20 × for 1 h at 1 min intervals with a Zeiss Axio Observer.

### Zebrafish imaging

Adult Tupfel longfin zebrafish were cared for by the Australian Zebrafish Phenomics Facility. Embryos 2.5 to 5 days post fertilisation carrying the BGUG transgene [51] for sparse mGFP labelling were used in the experiments. N-phenylthiourea (0.003%) kept the embryos transparent. Embryos were mounted in low melting point 1.5% agarose (SeaPlaque) in E3 medium on a glass coverslip and maintained at 28.5°C. Confocal image stacks were taken through the tectum (typically 40 to 60 µm, 1 µm intervals) and digitally flattened for analysis. GFP images at 10 and 1 min intervals were taken using a Zeiss LSM 510, through either a 20 × or 40 × objective. From the final time-lapse movies, spans 1 to 20 h long with visible growth cones were chosen for analysis.

### Semi-automated outline capture

All image processing was carried out using customised software developed using Matlab (Mathworks). To extract growth cone outlines, a quadrilateral region of interest was manually placed over the growth cone for selected frames of each movie and then automatically interpolated for intervening frames. Frames where growth cones were clearly bifurcating were excluded. Images within the region of interest were filtered using the Matlab image-texturing transforms *stdfilt* and *entropyfilt*. The growth cone outline was then manually optimised in approximately five to ten frames, and these candidate outlines were used to train linear support vector machines. This involved calculating local metrics (pixel intensity, size and distance from main body) pertaining to growth cone features that the user accepted or rejected (e.g., missing filopodia and foreign cell matter removal). The remaining movie frames were then automatically re-segmented using the support vector machines as a basis for attaching disconnected growth cone segments and features. All remaining imperfections were added or removed from the binary images via manual post-processing. These imperfections included particles floating in the media that had temporarily attached themselves to the growth cone, or defects in the underlying substrate (e.g., dark lines) that had become inappropriately incorporated into the growth cone outlines using the automated analysis. Additions to the growth cone occurred when the segmentation process failed to attach whole parts of the growth cone (e.g., when the axon became too thin to be segmented appropriately, or filopodia had clearly been missed). These judgements were made by the user performing the segmentation. Normally the total user interaction time required per movie was less than 1 h.

### Eigenshape analysis

All axon outlines were rotated to a common axis, and truncated such that their length (to the most distal tip of the growth cone) was 30 µm (20 µm for zebrafish data). For the pipette assay data, all growth cone outlines were mirrored about the *y*-axis such that the pipette tip was always located to the right of the growth cone. Coordinates of the growth cone representation used for the PCA (250 evenly spaced points) were determined by piecewise cubic-Hermite polynomial parametric representations (calculated using *pchip* in Matlab). PCA was performed using *princomp* in Matlab. The BIC was used to determine the number of PCA dimensions to retain. The last significant mode was taken as the first mode with negative BIC, given by

$$\begin{aligned} \text{BIC}_{k} &= N\sum_{i = 1}^{k} \log(V_{i}) + N(d-k)\log\left(\sum_{i = k+1}^{d} \frac{V_{i}}{d-k}\right)\\ &\quad + \left((d+1)k - \frac{k(k+1)}{2}\right) \log(N), \end{aligned} $$

where *k* is the number of modes to keep, *N* is the number of frames, *d* is the original dimensionality and *V* is the variance of each mode.

### Growth cone centre trajectories

The location of the growth cone centre (GCC) was estimated using a custom automated heuristic. The regions of the growth cone that had the darkest pixel intensity and that were more distant from the rotation base were picked as candidates for the GCC. If there were multiple candidate regions, the most likely was determined as the maximum of the transfer function $Y = (y^{h_{1}} \times I^{h_{2}})\phantom {\dot {i}\!}$ where *y* is the distance from the base of the rotated growth cone, *I* is the mean pixel intensity of the candidate group, and *h*_1_ and *h*_2_ were 1 and 2, respectively. The final GCC was calculated as the pixel weighted centroid of the candidate region. Clear anomalies in the automated method were corrected by manual manipulation.

### Trajectory analysis

GCC trajectories were smoothed using a moving average filter (step size of 3), discarding the first and last four frames to remove edge effects. Step length was measured as the Euclidean distance between successive time points, with average path length being the sum of the step lengths divided by the total number of time points. Average displacement was calculated as Euclidean distance between the first and last frames divided by the length of the movie. The growth cone bearing was calculated as the angle of each step, with the bearing change being calculated as the clockwise angle between two given time points. Initial angles for the pipette assay were calculated using the pipette location as a reference. For other assays the initial angle was calculated as the direction of movement over the first five frames. Final turning angles were measured as the angle formed between the initial direction and the direction of the GCC in the last frame from the GCC in the first frame.

### Statistical analysis of relationships

Unless otherwise stated all pairwise comparisons of data were performed using Wilcoxon rank-sum tests, while Kruskal–Wallis tests were used for comparisons of multiple groups. Relationships between individual mode scores versus trajectory statistics were calculated using Spearman correlations. Multiple linear regression analysis was used to correlate linear combinations of the mode scores with the trajectory statistics. When performing correlations and regression using oscillation statistics, only the first 2 h of movies were considered to remove bias based on movie length. The relationship between mode score oscillations to turning angle and average path length was calculated using both multiple linear regression and Spearman correlations.

### Time series analysis

A fast Fourier transform was calculated using *fft* in Matlab by centring the signal (mode score or growth cone area) about the mean and zero-padding the signal length to the next power of 2. The autocorrelation function was calculated using *autocorr* in Matlab. The extent of the oscillatory behaviour of the autocorrelation function was quantified using an oscillation strength metric adapted from [26]. In brief, the symmetric autocorrelation function was calculated and smoothed using a Gaussian kernel with a variance depending on imaging frequency. The central peak was truncated to the level of the next highest peak, and the fast Fourier transform calculated for the resultant signal. The final oscillation strength was defined as the power of the fast Fourier transform peak for the autocorrelation function divided by the mean power of the remaining frequencies.

### Computational model

The model was exactly as described in [40], except that there are two microtubules rather than one. In brief a microtubule of length *l*(*t*) grows according to

$$\frac{dl}{dt} = k_{a}[\!T] - k_{d} $$

and shrinks according to

$$\frac{dl}{dt} = -k_{s}, $$

where *t* is time, [ *T*] is the concentration of free tubulin, and *k*_*a*_, *k*_*d*_ and *k*_*s*_ are rate constants. The frequencies of catastrophe *f*_*c*_ (switch from the growth phase to the shrinkage phase) and rescue *f*_*r*_ (switch from the shrinkage phase to the growth phase) are given by

$$\begin{array}{@{}rcl@{}} f_{c} & = & a_{c}[\!T] + b_{c} \\ f_{r} & = & a_{r}[\!T] + b_{r} \end{array} $$

where *a*_*c*_,*a*_*r*_,*b*_*c*_ and *b*_*r*_ are constants. The values of all parameters were determined from experimental data [40], and are given in Table One of [40]. There was no spatial component to the model, and thus no time delay in the change in concentration available to one microtubule after binding or release of a tubulin monomer by the other microtubule. Simulations were performed using an event-based Monte Carlo approach as described in [40], coded in Matlab. Each simulation of 3×10^6^ events, corresponding to 2.6 h of real time for one growth cone, took about 3 min to run on a 2.8-GHz Intel Core i7 iMac.

## Abbreviations

BIC: Bayesian information criterion
GCC: growth cone centre
GFP: green fluorescent protein
M: mixed
NGF: nerve growth factor
PCA: principal component analysis
R: reflective
S: symmetric
SCG: superior cervical ganglion
